# QTL Dissection of Lag Phase in Wine Fermentation Reveals a New Translocation Responsible for *Saccharomyces cerevisiae* Adaptation to Sulfite

**DOI:** 10.1371/journal.pone.0086298

**Published:** 2014-01-28

**Authors:** Adrien Zimmer, Cécile Durand, Nicolás Loira, Pascal Durrens, David James Sherman, Philippe Marullo

**Affiliations:** 1 LAFFORT, Bordeaux, France; 2 Univ. Bordeaux, EA Œnologie 4577, ISVV, Villenave d'Ornon, France; 3 Center for Genome Regulation (FONDAP 15090007), University of Chile, Santiago, Chile; 4 CNRS UMR 5800, Univ. Bordeaux, INRIA project-team Magnome, Talence, France; University of Strasbourg, France

## Abstract

Quantitative genetics and QTL mapping are efficient strategies for deciphering the genetic polymorphisms that explain the phenotypic differences of individuals within the same species. Since a decade, this approach has been applied to eukaryotic microbes such as *Saccharomyces cerevisiae* in order to find natural genetic variations conferring adaptation of individuals to their environment. In this work, a QTL responsible for lag phase duration in the alcoholic fermentation of grape juice was dissected by reciprocal hemizygosity analysis. After invalidating the effect of some candidate genes, a chromosomal translocation affecting the lag phase was brought to light using de novo assembly of parental genomes. This newly described translocation (XV-t-XVI) involves the promoter region of *ADH1* and the gene *SSU1* and confers an increased expression of the sulfite pump during the first hours of alcoholic fermentation. This translocation constitutes another adaptation route of wine yeast to sulfites in addition to the translocation VIII-t-XVI previously described. A population survey of both translocation forms in a panel of domesticated yeast strains suggests that the translocation XV-t-XVI has been empirically selected by human activity.

## Introduction

Adaptation by natural selection occurs through the emergence of mutations that improve the fitness of an organism and its reproductive success in its environment. Identifying the genetic bases of adaptation is a great challenge in microbe genetics that may be carried out by different approaches including experimental evolution [1]–[3] and linkage analysis [3]–[8]. Diverse mutation types may impact trait variability and adaptation to an environment. In many cases, point mutations affecting transporters, transcription factors, and enzymatic activities produce variation in quantitative traits such as fitness, metabolite production, and gene expression level, as shown by many examples in yeast [3], [9]–[11] and bacteria [12]. Small insertions and deletions (INDEL) that generate frame shifts may affect also the integrity of proteins, causing drastic trait changes [3], [11], [13], [14]. Larger genome reorganizations may also drive the emergence of more adapted individuals. Yeasts, especially those belonging to the Saccharomyces genus, have been widely investigated for their genome plasticity and several examples of chromosomal rearrangements conferring a phenotypic advantage to individuals have been found [15]. Segmental duplications have been selected under laboratory [16], [17] or environmental conditions [18], [19]. Moreover, horizontal transfer of genetic material from eukaryotic [20], [21] or prokaryotic [22] origins has been brought to light. Recently, the physiological impact of some of them has been demonstrated for wine yeast [23]. Extra-copies of genes located in subtelomeric regions [24] as well as aneuploidies [25] are other examples of the role of yeast genomic plasticity in environmental adaptation. Another possible mechanism driving adaptation and evolution in yeast is chromosomal translocation [26]. This chromosomal rearrangement is initiated by a DNA double-strand break (DSB) that may generate both reciprocal and non-reciprocal translocations [27], [28] and that may be induced by physiological situations or by exogenous DNA damaging agents [26], [29], [30].

In yeast, translocation events are thought to play a role in adaptation and species evolution. For example, in the *Saccharomyces* clade reciprocal translocations may contribute with other mechanisms to hybrid sterility [31], [32]. Translocations may occur between small homologous sequences such as Ty elements, tRNAs, or microsatellites [2], [33], [34] and contribute to the karyotypic polymorphism found in yeast [31], [35], [36]. Such chromosomal rearrangements may have physiological consequences, affecting gene expression [19], [37]–[39], fitness [2], [19]and cell morphology [40]. Translocation events also play a role in environmental adaptation as described in wine yeast. Indeed a translocation between chromosome VIII and XVI increases sulfite resistance due to the creation of a new genetic environment regulating differentially the sulfite membrane pump, Ssu1p [37], [39], [41], [42].

The large number of chromosomal rearrangements found among yeast strains and their striking physiological consequences suggest that other unidentified translocation events may impact on the adaptation of wild yeast to their ecological niche. With the advent of next sequencing generation (NGS) chromosomal rearrangements can be more easily detected using specific approaches [43] or *de novo* assembling [26]. However, linking these events with a specific trait change remains a difficult task. In this work we describe the molecular dissection of a QTL controlling *lag phase*, the time necessary to start the alcoholic fermentation of grape must. In a previous study, this QTL was localized on the left arm of chromosome XV and was used to drive breeding strategies using molecular markers [44]. By analyzing the genomic sequence at this locus for both parental strains we bring to light a new reciprocal translocation between chromosome XV and chromosome XVI that confers an adaptive advantage to yeast strains in an enological context.

## Materials and methods

### Media, growth conditions and yeast strains used

All strains were grown at 30°C on YPD medium (1% yeast extract, 1% peptone, 2% glucose) solidified with 2% agar when required. When necessary the antibiotics G418 (Sigma-Aldrich, St Louis, Missouri, USA) and Nourseothricin (Werber BioAgent, Jena, Germany) were added at a final concentration of 100 µg/ml. The *Saccharomyces cerevisiae* strains used for QTL dissection are listed in Table 1. The panel of 48 strains used for PCR screening is presented in Table S1. For the QTL dissection of lag phase time the parents used (SB and GN) have been previously described [44].

**Table 1:** Yeast strains used.

Strain	Genetic background	Genotype	Origin
Y02160	*S288c*	BY4741; Mat a; his3Δ1; leu2Δ°; met15 Δ°; ura3Δ°; *YPL092w*::*kanMX4*	Euroscarf
Y01774	*S288c*	BY4741; Mat a; his3Δ1; leu2Δ°; met15 Δ°; ura3Δ°; *YOL083w*::*kanMX4*	Euroscarf
Y01865	*S288c*	BY4741; Mat a; his3 Δ 1; leu2 Δ°; met15Δ°; ura3Δ°; *YOL089c*::*kanMX4*	Euroscarf
Y03925	*S288c*	BY4741; Mat a; his3 Δ 1; leu2Δ°; met15Δ°; ura3Δ°; *HO*::*kanMX4*	Euroscarf
GN	monosporic clone of VL1	*HO/HO*; chr: VIII; chr: tXV-XVI;	Marullo *et al.* 2007b
SB	monosporic clone of BO213	*HO/HO*, chr:VIII, chrXV, chr:XVI	Marullo *et al.* 2007b
hoGN	GN	haploid derivate of GN, ho::NATMX4, mat a	Albertin *et al.* 2013
GΔ083	GN	*HO/HO*; *YOL083^GN^::kanMX4*/*YOL083^GN^::kanMX4*	this study
GΔ089	GN	*HO/HO*; *YOL089^GN^::kanMX4*/*YOL089^GN^::kanMX4*	this study
GΔ092	GN	*HO/HO*; *YPL092^GN^::kanMX4*/*YPL092^GN^::kanMX4*	this study
hoSB	SB	haploid derivate of SB, ho::kanMX4, mat α	Albertin et al. 2013
RG13	SB	haploid derivate of SB, ho::NATMX4, mat a	Richard Gardner (Auckland university)
SΔ083	SB	HO/HO; YOL083^SB^::kanMX4/YOL083^SB^::kanMX4	this study
SΔ089	SB	HO/HO; YOL089^SB^::kanMX4/YOL089^SB^::kanMX4	this study
SΔ092	SB	HO/HO; YPL092^SB^::kanMX4/YPL092^SB^::kanMX4	this study
BN	F1 hybrid	SB×GN hybrid	Marullo et al. 2007b
GΔS-083	BN	hemizygote hybrid *YOL083^GN^::kanMX4*/*YOL083^SB^*	this study
SΔG083	BN	hemizygote hybrid *YOL083^GN^/YOL083^SB^::kanMX4*	this study
GΔS089	BN	hemizygote hybrid *YOL089^GN^::kanMX4*/*YOL089^SB^*	this study
SΔG089	BN	hemizygote hybrid *YOL089^GN^/YOL089^SB^::kanMX4*	this study
GΔS092	BN	hemizygote hybrid *YPL092^GN^::kanMX4*/*YPL092^SB^*	this study
SΔG092	BN	hemizygote hybrid *YPL092^GN^/YPL092^SB^::kanMX4*	this study
F10-4A	monosporic clone of F10	YOL083^SB^	Marullo et al. 2009

### Spore dissection and haploid strain mating

Sporulation was induced on ACK medium (1% potassium acetate, 2% agar) after three days at 24°C. After incubation (1 h, 30°C) in a 2 mg/ml solution of cytohelicase (Sigma, Lisle d'Abeau Chesnes, France); spores were dissected by a micromanipulator Singer MSM Manual (Singer Instrument, Watchet, Somerset, UK) on YPD-agar. Hybrids were obtained by mixing haploid cells on YPD-agar for 6–18 h at 30°C; mating types were determined by using mating testers of both sex type.

### Genomic sequence analysis of the QTL region

Whole genome data sequences of strains SB and GN were obtained by using an Illumina pair end strategy. Briefly, genomic DNA was extracted from a saturated culture of 100 ml under anaerobic condition (YPD) using the genomic tip-100 kit (Qiagen, Courtaboeuf, FRANCE). Paired-end Illumina sequencing libraries were prepared from sonicated genomic DNA according to manufacturer protocols (Genomic DNA Sample Preparation) and were carried out by the Genomic and Transcriptomic facility of Bordeaux, FRANCE. Sequencing was performed on Illumina Genome Analyzer IIx (Illumina, CA) with a read length of 54 pb. The genome of both strains was first mapped on the reference genome using *stampy* program. The detection of SNP and short INDEL was carried out according to different criterions such alignment quality, read coverage, and genotype quality using the *SAMtools mpileup* [45] and *vcftools* programs [46]. The SNP and their relative effect of protein sequence were determined by *snpEff*. *De novo* assembly was then carried out using *mira3* [47] with 8 iterations. In this work we specially analyzed the contigs c343 and c23 of GN as well as c9 and c7 of SB containing the genes present in the dissected QTL. The annotated contig c343 and c23 of the strain GN containing the newly described XV-t-XVI translocation was deposited on EMBL database with the following study accession number: PRJEB4706.

### Genomic DNA extraction and PCR conditions

Genomic DNA extraction was carried out using the Genomic Wizard kit (Promega, Madison, Wisconsin, USA) or using FTA clone saver cards (Whatman, Maidstone, Kent, UK) according to manufacturer instructions. The PCR reactions were carried out on a BioRad machine (BioRad, Hercules, California, USA) using the Taq-&GO master mix (Qbiogene, Carlsbad, California, USA), in 20 µl final volume according to manufacturer conditions. All the PCR primers of this work are listed on Table S2 and were used at a final concentration of 0.5 µM. PCR fragment sizes were analyzed by agarose gel or by capillary electrophoresis with a multi NA apparatus (Shimadzu, Noisiel, FRANCE) using the 1000 pb gel kit.

### Gene deletion and hemizygous hybrids construction

The haploid derivate ho-SB and ho-GN were obtained by deletion of the *HO* locus using the *Kan-Mx4* and *Nat-Mx* cassettes, respectively [48]. Briefly, a deletion cassette was obtained by PCR using as template the genomic DNA of the strains Y03925 (Euroscarf, Franckfurt, Germany) or RG13 (kindly given by Professor Richard Gardner, Auckland, New Zeeland). The primers p25 and p26 allowed the amplification of a disruption cassette containing ∼500 bp of the flanking region of the *HO* locus. The deletions of *HAL9*, *ATG34* and *SSU1* were carried out following a similar strategy, using as template the genomic DNA of the strains Y01865, Y01774, Y02160, respectively. Primer couples p494/p495, p500/501, and p988/989 were used to amplify the deletion cassette. All the constructions were verified by both insertion and deletion PCR test. The insertion test consists in positively amplifying by PCR a fragment containing the 5′ part of the *KanMx4* cassette and ∼600 bp of the 5′-flanking region of the target gene. The deletion test consists in observing the absence of amplification of a central portion of the target gene. All the primers used for these tests are listed in Table S2. The parental strains SB and GN were transformed using the lithium acetate protocol described by Gietz and Schiestl [49]. Hemizygous hybrids were then constructed by classical genetics using tetrad microdissection and haploid mating techniques.

### PCR screening of translocations

To rapidly detect translocations involving the gene *SSU1*, PCRs tests were developped by designing appropriate primers (MWG Biotech, Germany). The translocations VIII-t-XVI and its reciprocal (XVI-t-VIII) were detected using the primers p764–p765 and p762–p763 respectively. The newly described translocation XV-t-XVI was detected using the primers P758–p765, while its reciprocal from (XVI-t-XV) was detected using the primers p760–761. The wild type copies of chromosomes VIII, XV and XVI were detected using the primers p786–p787, p758–p761, and p788–p789, respectively. Finally, an additional couple of primers was specifically designed for detecting wt-XVI chromosome (p788–p1032) and XV-t-XVI (p1031–p1032) in a Δ*SSU1* background. All primers amplified small genomic regions (<1000 bp) around the chromosomal break point. All the primer sequences are given on Table S2.

### Quantification of *SSU1* expression by qPCR

Extractions of mRNA and cDNA synthesis were carried out as previously described (Thibon et al. 2008). Briefly 1.10^7^ cells were harvested, washed and lysed using Fastprep FP120 apparatus (MP Biomedicals, Solon, Ohio). RNA was extracted using Tri reagent (Sigma, L'Isle d'Abeau Chesnes, France) and DNA contamination was treated using a DNA-free Kit (Ambion Inc., Austin, TX); RNAs were retrotranscribed into cDNAs using the iScriptTM cDNA Synthesis Kit (Bio-Rad, Hercules, CA). The extracted RNA was quantified using the ND-1000 UV-visible light spectrophotometer (NanoDrop Technologies, Wilmington, DE). The absence of contaminant genomic DNA in RNA preparations was verified using RNA as a template in real-time PCR assays. Each cDNA sample was analyzed two times independently by quantitative real-time PCR using iCycler iQ (Bio-Rad, Hercules, CA). ORF transcripts were amplified by using the following primers *SSU1*: p766/p767 (151 bp), *ALG9*: p605/p606 (163 bp), *ACT1*: p323/p904 (123 bp). The *ALG9* genes were used as a second reference gene as proposed previously [50]. Real-time quantitative PCRs (qPCRs) were carried out using the iQ SYBR Green Super Mix (Bio-Rad). Primers were added at a concentration of 0.3 mM each. The PCR program was as follows: 3 min at 95°C for initial denaturation, then 40 cycles of 10 s at 95°C, 30 s at TM and 30 s at 72°C. A final melt curve was carried out for control specific amplification by 36 cycles of 10 s starting at 65°C, with increasing steps of 0.5°C at each cycle. The PCR efficiencies were 80.2%, 89.7%, 88.7% for *SSU1*, *ACT1* and *ALG9*, respectively. A standard curve was determined for each gene, where x is the threshold cycle and y is the log value of the starting quantity (ng): *SSU1* (y = −3.90x+21.44, adjusted R^2^ = 0.995); *ACT1* (y = −3.62x+22.44, adjusted R^2^ = 0.985); *ALG9* (y = −3.65+23.81, adjusted R^2^ = 0.994. Standard curves were obtained from eight points in triplicate and linearity was observed from 0.0366 ng to 183 ng of DNA. A threshold value for the fluorescence of all samples was set manually, to maintain the same value in each experiment. The relative amounts of *SSU1* with respect to each reference gene were calculated from the standard curve. As similar results were obtained from both reference gene only *SSU1/ACT1* are graphically presented.

### Measure of lag phase time during alcoholic fermentation

Fermentations were carried out using the model synthetic medium (KP-medium) as previously described [51]. Pre-cultures were run for 24 h at 24°C under orbital agitation (150 rpm) in the fermentation media filtered through a 0.45 µm nitrate-cellulose membrane (Millipore, Molsheim, France) and diluted 1∶1 with milli-Q water. The inoculum concentration was 10^6^ viable cells *per* ml. Fermentations were run in closed 150 mL glass-reactors, locked to maintain anaerobiosis, with permanent stirring (300 rpm) at 24°C. The SO_2_ amount was adjusted in the fermentation media but not in the preculture at a concentration ranging from 0 to 40 mg/L as specified in the text. The CO_2_ released was monitoring by measurement of glass-reactor weight loss regularly and expressed in g L^−1^. The raw fermentation kinetics data were smoothed by a Loess function allowing the estimation of the *lag phase time* (h) that was the time between inoculation and the release of 3 g/L of CO_2_. All the fermentations were done in triplicate.

### Cell concentration and viability monitoring

During the lag phase period, bioreactors were sampled and yeast populations were analyzed using a flow cytometer (Quanta SC MPL, Beckman Coulter, Fullerton, California), equipped with a 488 nm laser (22 mW) and a 670 nm long-pass filter. Samples were diluted in McIlvaine buffer pH = 4.0 (0.1 M citric acid, 0.2 M sodium phosphate dibasic) and propidium iodide (0.3% v/v) was added in order to stain dead cells (FL3 channel). ***Viability*** and ***cell concentration*** were expressed in %, and cells per ml respectively. In order to measure the physiological change of yeast during the lag phase the ***LP viability*** was calculated as follow:

(Vmin-Vp)/Vp. Were Vp and Vmin are the viability of the preculture and minimal viability value observed during the lag phase. Indeed, negative values of LP viability indicate that yeast cells underwent a drop of viability during the lag phase.

### Analytical methods

The total SO_2_ and free SO_2_ (mg/L) were measured by Pararosaniline titration [52] by the analytical laboratory SARCO (Bordeaux, France).

### Statistical and graphical analyses

All the statistical and graphical analyses were carried out using the R program. The variation of each trait analyzed was estimated by an analysis of variance (ANOVA) according to the model described in the result section. For each variable, the homogeneity of the variance was assessed using a Levene test by means of R's *car* package version 2.15.1 [53], as well as the normality of residual distribution using a Shapiro test [53]. Duncan's multiple comparison was used to determine which means differ significantly (Duncan's multiple comparison, p<0.05) using the *agricolae* package. When required, pairwise comparisons were carried out using the Wilcoxon test with at least 5 independent repetitions.

## Results

### QTL dissection assisted by whole genome sequencing reveals a translocation event between the chromosome XV and XVI

**Figure 1:**
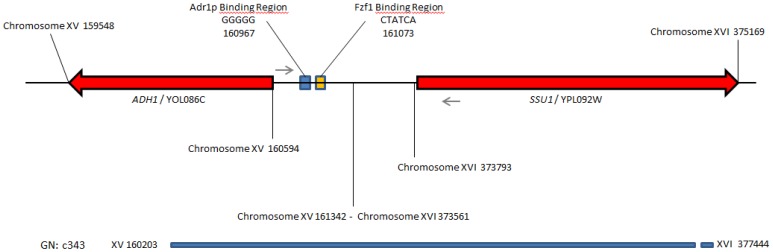
Description of the XV-t-XVI translocation. The diagram represents the breakpoint of translocation XV-t-XVI for the strain GN. The genomic region of contig c343 includes the 5′ portion of gene *ADH1* and the coding sequence of *SSU1* (red arrows) and their respective regulating regions Adr1p and Fzf1 binding regions (yellow diamond). The small arrows represent the primers p758 and p761 allowing identifying by PCR the XV-t-XVI translocation.

**Figure 2:**
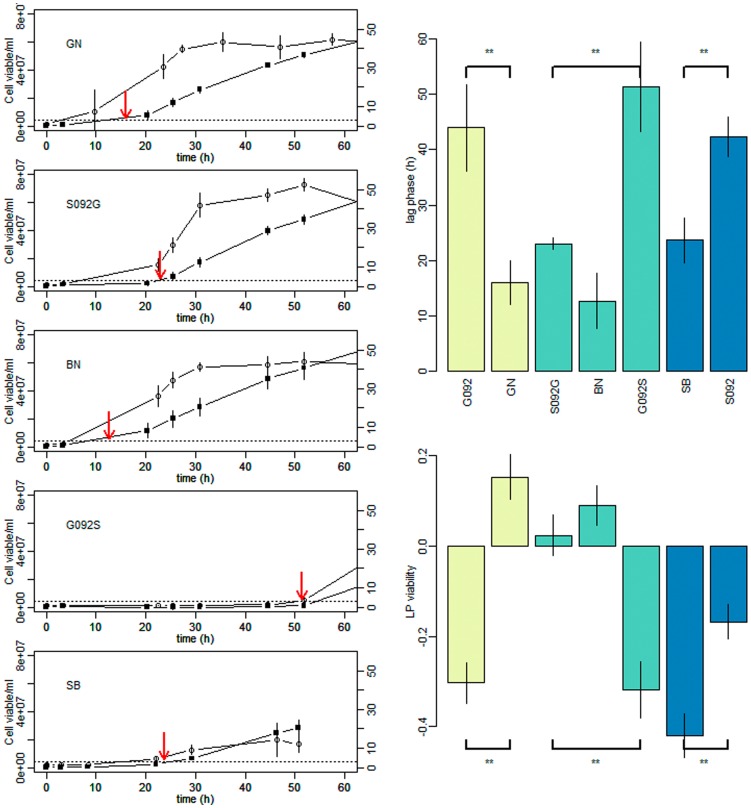
The *SSU1^GN^* allele impact the lag phase in BN background. On the left panel, the time course of viable cell concentration (open circle) and the CO_2_ production (black square) during the 60 first hours after yeast inoculation are shown. Data presented are the mean of three independent repetitions with standard error for parental strains (SB and GN), F1 hybrid (BN) as well as hemizygous hybrids (GO92S, S092G) for the gene *SSU1* (*YPL092w*). The lag phase time computed is indicated by a red arrow. The bar plots on the right panel represent mean values and standard error measured for 3 independent repetitions for the lag phase (h) and the LP viability. These traits were measured for parental strains (SB and GN), F1 hybrid (BN), the hemizygous hybrids (GO92S, S092G) for the gene *SSU1* (*YPL092w*) and the *SSU1* double deletion mutant of GN and SB (G092 and S092). Isogenic strains are represented with the same color, GN = light green, BN = green, SB = blue. The statistic difference between isogenic strains was tested using a Wilcoxon test. The significant level is coded **, for p-values<0.01.

In a previous work [44] we identified by QTL mapping a major locus controlling the time necessary to initiate the alcoholic fermentation in winemaking conditions (lag phase). This QTL named QTL-XV was narrowed between the gene *HAL9* (*YOL089c*) and *ATG19* (*YOL082w*) in a region of nearly 20 kb containing 8 genes. The aim of this work is to dissect this QTL and find the genetic polymorphisms explaining the phenotypic difference between the parental strains SB and GN. By sequencing the whole genome of parental strains using an Illumina pair end strategy, between the parental strains, 33 Single Nucleotide Polymorphisms were detected in this region generation fifteen non synonymous mutations affecting five genes (Table S3). Putative deleterious mutations were found for two of them (*HAL9* and *ATG34*) and we tested their impact on lag phase by reciprocal hemizygosity assay (RHA) according to Steinmetz *et al.* [6] (Figure S1 and S2). This first tentative failed to identify the cause of lag phase difference suggesting the role of other genetic modifications such as gross chromosomal rearrangements. To identify such modifications, the genomes of parental strains SB and GN were *de novo* assembled generating between 360 to 400 contigs per strains (Table S4). We used as query sequence, the 20 kb region of QTL-XV of the strain S288c and we carried out a *blastn* analysis against the parental set of *de novo* contigs. For the strain SB the entire sequence match with one contig showing the same syntheny than S288c strain. In contrast for the parental strain GN, we found evidence for a reciprocal translocation between the chromosome XV and the chromosome XVI covered by the contigs c343 and c23. The translocation event was localized between the coding sequences of *ADH1* and *PHM7* (chromosome XV) and between the coding sequence of *SSU1* and *NOG1* (chromosome XVI). The exact chromosomal break point occurs at the position 161342 and 373561 for the chromosome XV and XVI, respectively, as confirmed by PCR sequencing. This break point occurs in a low complexity AT rich region without evident homology between chromosomes. As shown in Figure 1, this chromosomal rearrangement generates one XV-t-XVI chromosome, remodeling the promoter environment of the *SSU1* gene. This gene encodes for a plasma membrane protein playing a major role in sulfite detoxification. The expression regulation of *SSU1* has been widely investigated in the past and depends on the transcription factor Fzf1p, a protein subjected to positive selection within *Saccharomyces* species [54] and among wine *S. cerevisiae* strains [42]. In the translocation XV-t-XVI described in this work, the start codon of *SSU1* is then located at 501 bp of an Fzf1p binding site (CTATCA) and at 607 bp of an Adr1p binding site (GGGGG) known to activate the transcription of alcohol dehydrogenase during fermentation conditions [55]. The presence of these promoter regions suggest that the expression of this gene might be enhanced. The role of the *SSU1* gene in lag phase was further investigated by analyzing the time course of cell growth and CO_2_ production during the first hours of alcoholic fermentation by RHA. As shown in Figure 2, the *SSU1^GN^* allele allowed initiating the fermentation 28.3 h sooner than its *SSU1^SB^* counterpart (Wilcoxon test, p<0.01). Interestingly, the reduction of the *SSU1* copy number in the BN hybrid has an effect, and both hemizygous hybrids showed a longer lag phase than control. This means that both *SSU1* alleles (GN and SB) play an active role in starting alcoholic fermentation, but that the *SSU1^GN^* allele is more efficient than *SSU1^SB^*. Physiologically, the lag phase difference observed was in part explained by yeast viability, which is significantly higher when the GN allele was present (74% vs 59%). However the relation between lag phase and viability is not simple, the parent SB initiated the fermentation much more quickly than the G092S hybrid, despite its lower viability. All these findings strongly suggested that the *SSU1^GN^* allele is the main cause of lag phase discrepancy between strains GN and SB and validated the QTL dissection at a gene level.

### Impact of two chromosomal translocations involving *SSU1* on lag phase

**Figure 3:**
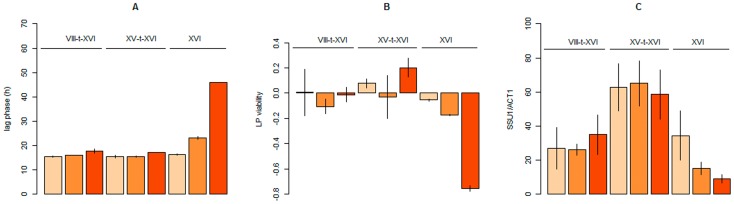
Effect of translocations XV-t-XVI and VIII-t-XVI on lag phase, LP viability and *SSU1* expression. The lag phase, LP viability, and expression level of *SSU1* gene are shown in panel A, B and C, respectively. Data were obtained from alcoholic fermentation using the strains F10, GN and SB carrying the chromosome forms VIII-t-XVI, XV-t-XVI and nt-XVI, respectively. For each strain, fermentations were carried out in synthetic media containing increasing concentrations of total SO_2_ (0, 20 and 40 mg), graphically represented by orange scale 0 mg = light orange, 20 mg = orange, 40 mg = dark orange. For panel A and B, bar plots represent the mean with standard error of three independent repetitions. For panel C the expression level of *SSU1* was normalized by *ACT1*. Data represent the mean and standard error of 6 to 9 samples collected during the lag phase.

The Ssu1p sequence of SB and GN were compared with those of 30 *Saccharomyces cerevisiae* yeasts. Between GN and SB, five single amino-acid polymorphisms were found (F16S, V19M, A122S, I151V, R291K). The substitutions F16S and I151V were found only for the SB strains while other SAPs have allelic frequencies ranging from 0.1 to 0.6 among the 30 sequences investigated. However, no deleterious effect of these SAPs were found using the SIFT algorithm (α = 0.05) [56] by aligning these SAP with 39 eukaryote sulfite pump sequences (data not shown). In addition, the comparison of S092 with SB and GN with G092 strains demonstrated that in both parents Ssu1p is functional and able to reduce lag phase in the presence of SO_2_. This evidence suggests that the reorganization of the *SSU1* promoter region due to the translocation XV-t-XVI, rather the function of the Ssu1 protein, might be the cause of the impact of the *SSU1^GN^* allele. Interestingly, a translocation event implying the *SSU1* gene with the promoter region of *EMC34* has been described for wine yeast [41]. This translocation concerns chromosomes VIII and XVI (VIII-t-XVI) [41], allowing the formation of the so-called *SSU1-R* allele [57] and increasing sulfite tolerance by enhancing *SSU1* expression level [37], [39], [58]. In order to compare the effect of the new XV-t-XVI translocation described here with the VIII-t-XVI and the wild type forms (nt-XVI), lag phase duration was investigated in a synthetic must containing different amounts of total SO_2_ (0, 20 and 40 mg/L) using the strains GN (XV-t-XVI), F10 (VIII-t-XVI) and SB (nt-XVI). 40 mg/L SO_2_ correspond to the standard concentration found in wine making conditions at the beginning of alcoholic fermentation [59]. The lag phase was monitored by weight loss each 2 hours and culture samples were regularly taken at different times since the alcoholic fermentation started. Cell growth and viability were monitored by flow cytometry and a transcriptional analysis of *SSU1* was carried out during the lag phase period. The Table 2 summarizes the analysis of variance carried out; the Figure 3 shows the *lag phase*, *lag viability*, and *SSU1 expression level* for all the strain-media conditions. As expected and previously reported in winemaking conditions, lag phase time is impacted by SO_2_ concentration in the medium [60], [61]. However strains harboring translocations XV-t-XVI and VIII-t-XVI are much less affected than the strain SB and initiated the fermentation after 18 hours even in presence of 40 mg of total SO_2_. At this concentration, the strain SB showed a much longer lag phase (46 hours) (panel A). The behavior of SB can be explained by cellular viability during the lag phase. As shown on panel B the *lag viability* of SB is drastically affected when SO_2_ concentration increases, while other strains harboring a translocation form are not significantly affected. In presence of 40 mg/L of SO_2_ the viability of strain SB decreases up to 80% of its initial value, explaining the long lag phase observed for this strain in this condition. These results suggest a toxic effect of SO_2_ on the strain SB, explaining the lag phase discrepancy observed in the BN progeny. Regarding the expression levels of *SSU1*, no induction by SO_2_ can be detected (Table 2) as previously reported for other wine yeast [37]. In contrast, as shown on panel C, a clear translocation effect was detected by variance analysis whatever the SO_2_ concentration in the medium (ANOVA, p<0.001). The strain GN (XV-t-XVI) showed a 3 fold increased expression of *SSU1* compared to other strains during the lag phase while the strains SB (nt-XVI) and F10 (VIII-t-XVI) cannot be statistically discriminated from each other (Duncan test, α = 0.01). This result suggested that *SSU1* expression level during the lag phase is the cause of trait discrepancy between SB and GN. Despite no global strain effect being detected between F10 and SB, a significant difference (3.7 fold) was found between F10 (VIII-t-XVI) and SB (nt-XVI) when the SO_2_ concentrations was increased up to 40 mg/L (Wilcoxon, p<0.05) indicating that in high SO_2_ concentration the strain F10 may export more efficiently the SO_2_ than the strain SB, subsequently reducing the lag phase.

**Table 2:** Analysis of variance of lag phase, viability change, and *SSU1* expression according to the translocation and total SO_2_ concentration in the medium.

	*Lag phase (h)* a		*LP Viability* a		*SSU1/ACT1* b	
	part of variance	p value	part of variance	p value	part of variance	p value
Translocation	36.4	<0.001	36.2	<0.001	28.4	<0,001
SO_2_	22.2	<0.001	8.0	<0.05	0.5	ns
Translocation:SO_2_	34.9	<0.001	26.4	<0.01	2.3	ns
qPCR block	nd		nd		8.0	<0.05
Residual	6.4		29.4		60.8	

a the model used was *Z1* = *μ*+*translocation*_(i,j,k)_*SO_2(i,j,k)_ *+ε*_i_, which allows the estimation of the part of the variance explained by the chromosome XVI forms (n = 3) and the SO_2_ concentration (n = 3). For each condition, 3 repetitions were analyzed. nd = not determined.

b the model used was *Z2* = *μ*+*translocation*_(i,j,k)_*SO_2(i,j,k)_+qPCR_(i,j)_ *+ε*_i_, which allows the estimation of the part of the variance explained by the chromosome XVI forms (n = 3), the SO_2_ concentration (n = 3), and the qPCR blocks. For this model 6 to 9 repetitions were analyzed for each conditions depending on the lag phase time. ns = not significant.

### Landscape of XV-t-XVI and VIII-t-XVI translocations among industrial yeast strains

**Figure 4:**
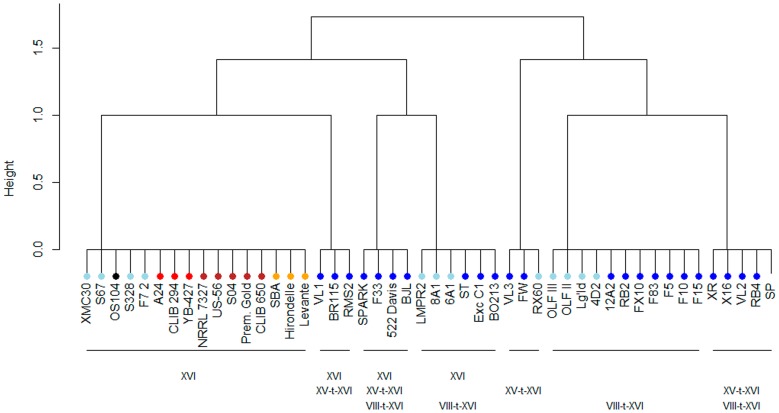
Distribution of translocations XV-t-XVI and VIII-t-XVI among industrial yeast on different origin. The Euclidian distance among 48 yeast strains according to the presence of the three allelic forms on chromosome XVI (nt-XVI, VIII-t-XVI, and XV-t-XVI) was calculated using the R program. The origin of each yeast strain used is coded by a color point. Light blue = grape isolates, dark blue = wine starters, black = natural isolate, red = distillery, brown = brewery, orange = bakery.

**Figure 5:**
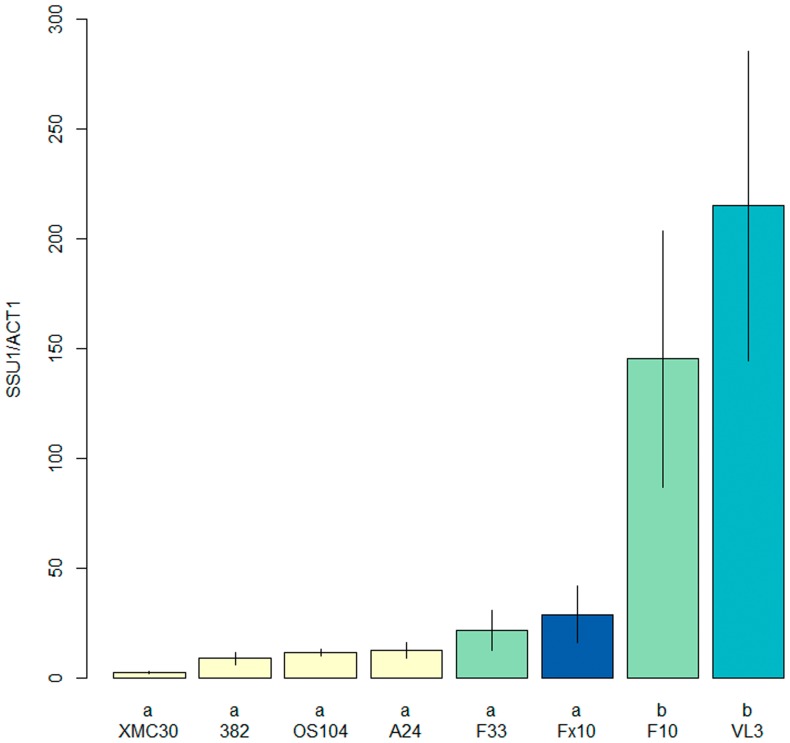
Expression level of *SSU1* at mid-alcoholic fermentation according to the chromosome XVI forms. The *SSU1* expression level was measured during the alcoholic fermentation. Yeast cultures were sampled at 40 g/L of CO_2_ at the end of the growth phase. Bar plots represent the mean and the standard error of three independent extractions for eight strains (XMC30, 382, 0S104, A24, F33, Fx10, VL3). The chromosome XVI forms of the strains are coded by grey tones: Yellow, light green, dark green and dark blue represent respectively strains with only nt-XVI, only VIII-t-XVI, only XV-XVI and with both VIII-t-XVI and XV-t-XVI forms. The letters on the bottom of strain name represent the statistical groups computed by Duncan analysis of the one-way ANOVA model used.

In order to identify rapidly the various forms of chromosome XVI described in this work, a set of PCR based tests were set up as described in methods. The occurrence of the newly described form of translocation XV-t-XVI within a panel of 48 yeast strains from different origin was then investigated (Table S1). To test if this chromosomal rearrangement is a rare event or a natural selection outcome, a panel of 24 commercial starters was compared with 12 natural grape isolates, 3 distillery strains, 5 brewery strains, 3 bakery strains and one oak isolate. The distribution of the three forms of chromosome XVI is shown in Figure 4. Yeast strains isolated form other industries (bakery, brewery and distillery) as well as the natural isolate OS104 did not possess translocated form of chromosome XVI. By contrast, almost wine yeasts (32/36) showed at least one translocation involving the *SSU1* gene (XVI-t-XVI or VIII-t-XVI). In a few cases, at least three copies of chromosomes XVI were present in the yeast genome, confirming the aneuploidy nature of many wine yeasts [35]. Strikingly, only the industrial starters (16/24) have at least one copy of XV-t-XVI in their genome, suggesting that this specific translocation has been selected by humans within the best performing individuals. The already described VIII-t-XVI form was found in 26 wine strains including 18 starters and constitutes the major allelic form of chromosome XVI in the wine group. The fact that many commercial strains, selected for their fermentation performances, carry the XV-t-XVI form suggests that this chromosomal rearrangement must be considered as the consequence of anthropic selection to improve the fermentation capacities of yeast. To test the impact of the different chromosomes of type XVI on *SSU1* expression, an additional experiment was carried out at 40% of CO_2_ released in a synthetic medium. Of the eight strains used, four have wild-type copies of chromosome XVI (nt-XVI) (382, A24, XMC30 and OS104), two carry the VIII-t-XVI translocation (F10, Fx10), one has the XV-t-XVI translocation (VL3), and the last possesses all the possible forms (F33). As shown in Figure 5, the strains carrying translocations have the highest expression values for *SSU1*; however, from a statistical point of view, only the strains VL3 and F10 expressed significantly more *SSU1* than the nt-XVI group. The strain F33, despite the presence of the two types of translocation forms, has a *SSU1* expression level identical to the wild type form. Altogether, these results suggest that other genetic determinisms may have an epistatic control on the *SSU1* expression during the alcoholic fermentation in some genetic backgrounds.

## Discussion

### QTL dissection: searching for SNPs, finding a translocation

Over the ten past years, *Saccharomyces cerevisiae* has become an excellent model of quantitative genetics in order to unravel the complex determinism of natural trait variation [4]. Indeed, QTL mapping approaches match perfectly with the intrinsic characteristics of budding yeast: huge genetic and phenotypic variation, short life cycle, genome compactness, high recombination rate, and molecular genetics facilities. The advent of this new yeast genetics discipline is due to decisive technological breakthroughs that provide numerous markers in single experiments, such DNA microarrays [3], [9], [10], [62]–[65] and NGS approaches [5], [8], [66]–[68]. In these numerous studies, markers were obtained by physically mapping genetic polymorphisms detected within strains onto a reference strain genome (S288c). In many cases this approach was justified by the fact that one of the parental strains was the laboratory strain itself. This avoids the reconstitution of the true genetic map, assuming that the parental strains genomes are collinear. However, QTL programs can be carried out with two [9], [69] or more natural strains [66], [68] that might have a different chromosomal architecture than the reference genome. In our study we first mapped the SNP found between the parental strains GN and SB, onto the chromosomal organization of S288c and we invalidated our candidate genes by an RHA. In the present case, this strategy was elusive since the genetic modification involved in lag phase was a translocation affecting the expression level of the *SSU1* gene. The existence of a reciprocal translocation at this locus was previously suggested by analyzing the germination rate of backcrossed lines [44]. However, on the basis of the microarray data that were collected, the linkage between chromosome XV and chromosome XVI was undetectable (data not shown). Using *de novo* assembly of pair-end reads, we identified in the parental strain GN a new translocation between chromosomes XV and XVI that explains most of phenotypic variation in the BN hybrid progeny. To our knowledge, this is the first example of QTL dissection that demonstrates the impact of a gross chromosomal rearrangement on phenotypic trait variation. This work also highlights the importance of using *de novo* assembly rather than mapping assembly for dissecting QTLs with natural yeast. This point is crucial when the parental strains are derived from industrial backgrounds, which are known to carry many chromosomal rearrangements and aneuploidies [15].

### Convergent evolutionary routes affect the *SSU1* expression level

Sulfite resistance has been widely documented in yeast and in particular for the winemaking context as recently reviewed by Divol et al [70]. After diffusing in the cell by passive transport [71] under its molecular form (SO_2_·H_2_O), sulfite interacts with yeast metabolism mainly under its bisulfite ion form (HSO_3_^−^). Sulfites inhibit key glycolytic enzymes like Tdh and Adh proteins, bind carbonyl [72] compounds such pyruvate and acetaldehyde, affect transporter activity by binding membrane proteins [70], and down-regulate the expression of many central metabolism genes [73]. These multiple targets explain why sulfite resistance is a quantitative trait [74] involving different detoxification routes such as sulfite export [42], acetaldehyde accumulation [75] and the sulfur reduction pathway [76]. The most efficient detoxification mechanism is sulfite efflux by the Ssu1p pump. The molecular mechanisms modifying the activity and expression of this transporter have been widely investigated: punctual mutations [77], differential gene expression [42], [57], as well as possible post-transcriptional regulations of *SSU1* [37], are the main molecular mechanisms identified.

Here we have described a new allelic variation widely spread among wine yeasts that increases the *SSU1* expression level by modifying drastically the upstream region of *SSU1*. The relative distance of the consensus binding sites of Adr1 (−607 pb) and Fzf1p (−501 bp) from the *SSU1* start position closely matches with the optimal position of these regulatory sites [78]. To date, all the natural allelic variations explaining the adaptation of wine yeast to sulfite modulate directly or indirectly the expression level of *SSU1* gene. Interestingly, other wine microbes such as the bacterium *Oenococcus oeni* seem to have a gain in fitness when carrying a sulfite pump (*tauE*) similar to *SSU1* [79]. This convergence indicates that sulfite pumping over is the most efficient way for a microorganism to adapt to sulfite by natural selection. These mutations can be classified into two groups. First, a *trans*-acting group, that affects both the activity [42], [54], and expression level [54] of Fzf1p and by consequence could impact the expression level of *SSU1*. Interestingly, comparative genomics within the *Saccharomyces* clade demonstrated that this gene is under strong positive selection [54]. Second, a *cis*-acting group characterized by two independent translocation events VIII-t-XVI [41] and XV-t-XVI (this study) is highly prevalent in wine yeast population.

### Adaptative role of translocations in yeast

The role of translocations in yeast adaptation have been widely described for the *Saccharomyces* clade but also for other yeast genera [80] and pathogenic fungi [81]. Within Saccharomyces species, translocations contribute with other mechanisms to reduce the fertility at a post zygotic level [31], [32]. At the intraspecific level, translocations are thought to be a rapid way to gain an adaptive mutation by creating a new chromosomal environment [15]. Moreover translocations are more stable than collinear segmental duplications and may confer a more stable gain of fitness [82]. In a laboratory environment, many examples demonstrate the adaptive benefit of translocation under nutrient limitation [2], [83] or inhibitory conditions [84]. However, reports of translocation events occurring in a natural population and conferring a selective advantage to environmental conditions are rare. For a long time, the translocation event VIII-t-XVI reported by [41] was the unique example of a translocation in natural yeast population. Recently another example of translocation between the chromosomes VII and VIII associated with segmental duplications has been reported for a *Saccharomyces cerevisiae* population adapted to high copper concentrations. The new example of translocation described here confirms that chromosomal translocations are an important adaptive route in harsh environmental conditions. Moreover we demonstrated here that both translocations VIII-t-XVI and XV-t-XVI conferred a selective advantage by shortening by many hours the growth lag phase in a medium containing SO_2_. These conditions match better to the natural conditions than the classical toxin resistance test carried out by plating yeast on YPD. A competition assay was performed with strains carrying XV-t-XVI or nt-XVI inoculated at the same rate. The strains carrying XV-t-XVI translocation clearly outcompeted their non-translocated counterparts, as more than 95% of colonies carried the XV-t-XVI chromosome form at the end of the fermentation, demonstrating the translocation advantage in only five generations (data not shown). The translocation benefit was also confirmed by their prevalence in wine yeast (both grape isolate and industrial starters). These strains may have been directly or indirectly selected by human activity for their rapid colonization of grape must containing sulfur dioxide. Interestingly, the commercial starter group is the unique one carrying the translocation XV-t-XVI. Further experiments could test the relative advantage between both translocations forms in isogenic conditions.

### Concluding remarks

In this study, we elucidated the molecular basis of a QTL controlling a fermentation trait that explains the adaptation of yeast strains to human activity (*i.e.* winemaking in presence of sulfite). For the first time the nature of genetic variation elucidated was not here a SNP or a small INDEL but a newly described chromosomal rearrangement creating a “gain of function” allele. Indeed most of the QTL dissected here brought to light deleterious alleles in one of the parental strain affecting the function or the expression of a protein. Finally, we demonstrated that the translocation XV-XVI form is present in many yeast strains used in industry that are likely selected for their ability to rapidly colonize grape must.

## Supporting Information

Figure S1 **The time course of viable cell concentration (open circle) and the CO_2_ production (black square) during the 60 first hours after yeast inoculation are shown.** Data presented are the mean of three independent repetitions with standard error for parental strains (SB and GN), F1 hybrid (BN) as well as hemizygous hybrids (G083S, S083G, G089S and S089G) for the gene *ATG34* (*YOL083w*) and *HAL9* (*YOL089c*) respectively. The lag phase time computed is indicated by a red arrow.
(TIF)

Figure S2 **Reciprocal heterozygosity analysis of** ***ATG34*** **and** ***HAL9*** **for lag phase.** The lag phase (h) as well as the LP viability was measured for parental strains (SB and GN), F1 hybrid (BN) as well as hemizygous hybrids (G083S, S083G, G089S,S089G) for genes *ATG34* (YOL083w) and *HAL9* (YOL089c). The bar plots represent mean values and standard errors measured for 3 independent repetitions. The statistic difference between isogenic strains was tested using a Wilcoxon test. Isogenic strains are represented with the same color, GN = light green, BN = green, SB = blue. The significant levels was coded *, and **, for p-values<0.05 and <0.01 respectively.
(TIF)

Table S1 (DOCX)

Table S2 (DOCX)

Table S3 ^a^ The SNPs and SAPs presented are given according to the reference genome (S288c strain) http://www.yeastgenome.org. ^b^ SIFT analysis was carried out on the web site http://sift.jcvi.org/ using homologous proteins identified by blastp http://blast.ncbi.nlm.nih.gov; the number of proteins aligned varied between 6 to 35 according to the protein tested with a pvalue cut off equal to 0.01.
(DOCX)

Table S4 Reads were done in pairs. See Materials and Methods for description of the assembly process. Coverage was computed on the basis of the S288c genome size (12071326 nt), obtained from Saccharomyces Genome Database.
(DOCX)

## References

[1] Elena SF, Lenski RE (2003) Evolution experiments with microorganisms: the dynamics and genetic bases of adaptation. Nat Rev Genet 4: 457–469. 12776215 10.1038/nrg1088

[2] Dunham MJ, Badrane H, Ferea T, Adams J, Brown PO, et al (2002) Characteristic genome rearrangements in experimental evolution of *Saccharomyces cerevisiae* . Proc Natl Acad Sci U S A 99: 16144–16149. 12446845 10.1073/pnas.242624799 PMC138579

[3] Romano GH, Gurvich Y, Lavi O, Ulitsky I, Shamir R, et al (2010) Different sets of QTLs influence fitness variation in yeast. Mol Syst Biol 6. 10.1038/msb.2010.1 PMC2835564 20160707

[4] Liti G, Louis EJ (2012) Advances in quantitative trait analysis in yeast. PLoS Genet 8: e1002912. 22916041 10.1371/journal.pgen.1002912 PMC3420948

[5] Yang Y, Foulquie-Moreno MR, Clement L, Erdei E, Tanghe A, et al (2013) QTL Analysis of High Thermotolerance with Superior and Downgraded Parental Yeast Strains Reveals New Minor QTLs and Converges on Novel Causative Alleles Involved in RNA Processing. PLoS Genet 9: e1003693. 23966873 10.1371/journal.pgen.1003693 PMC3744412

[6] Steinmetz LM, Sinha H, Richards DR, Spiegelman JI, Oefner PJ, et al (2002) Dissecting the architecture of a quantitative trait locus in yeast. Nature 416: 326–330. 11907579 10.1038/416326a

[7] Salinas F, Cubillos FA, Soto D, Garcia V, Bergstrom A, et al (2012) The genetic basis of natural variation in oenological traits in Saccharomyces cerevisiae. PLoS One 7: e49640. 23185390 10.1371/journal.pone.0049640 PMC3504119

[8] Ehrenreich IM, Torabi N, Jia Y, Kent J, Martis S, et al (2010) Dissection of genetically complex traits with extremely large pools of yeast segregants. Nature 464: 1039–1042. 20393561 10.1038/nature08923 PMC2862354

[9] Marullo P, Aigle M, Bely M, Masneuf-Pomarede I, Durrens P, et al (2007) Single QTL mapping and nucleotide-level resolution of a physiologic trait in wine Saccharomyces cerevisiae strains. FEMS Yeast Res 7: 941–952. 17537182 10.1111/j.1567-1364.2007.00252.x

[10] Brem RB, Yvert G, Clinton R, Kruglyak L (2002) Genetic dissection of transcriptional regulation in budding yeast. Science 296: 752–755. 11923494 10.1126/science.1069516

[11] Araya C, Payen C, Dunham M, Fields S (2010) Whole-genome sequencing of a laboratory-evolved yeast strain. BMC Genomics 11: 88. 20128923 10.1186/1471-2164-11-88 PMC2829512

[12] Zhang Q, Lambert G, Liao D, Kim H, Robin K, et al (2011) Acceleration of Emergence of Bacterial Antibiotic Resistance in Connected Microenvironments. Science 333: 1764–1767. 21940899 10.1126/science.1208747

[13] Doniger SW, Kim HS, Swain D, Corcuera D, Williams M, et al (2008) A Catalog of Neutral and Deleterious Polymorphism in Yeast. PLoS Genet 4: e1000183. 18769710 10.1371/journal.pgen.1000183 PMC2515631

[14] King T, Seeto S, Ferenci T (2006) Genotype-by-Environment Interactions Influencing the Emergence of rpoS Mutations in Escherichia coli Populations. Genetics 172: 2071–2079. 16489226 10.1534/genetics.105.053892 PMC1456365

[15] Liti G, Louis EJ (2005) Yeast evolution and comparative genomics. Annu Rev Microbiol 59: 135–153. 15877535 10.1146/annurev.micro.59.030804.121400

[16] Dunham MJ, Badrane H, Ferea T, Adams J, Brown PO, et al (2002) Characteristic genome rearrangements in experimental evolution of Saccharomyces cerevisiae. Proceedings of the National Academy of Sciences 99: 16144–16149. 10.1073/pnas.242624799 PMC138579 12446845

[17] Koszul R, Caburet S, Dujon B, Fischer G (2004) Eucaryotic genome evolution through the spontaneous duplication of large chromosomal segments. Embo J 23: 234–243. 14685272 10.1038/sj.emboj.7600024 PMC1271662

[18] Welch JW, Fogel S, Cathala G, Karin M (1983) Industrial yeasts display tandem gene iteration at the CUP1 region. Molecular and Cellular Biology 3: 1353–1361. 6621529 10.1128/mcb.3.8.1353-1361.1983 PMC369981

[19] Chang SL, Lai HY, Tung SY, Leu JY (2013) Dynamic large-scale chromosomal rearrangements fuel rapid adaptation in yeast populations. PLoS Genet 9: e1003232. 23358723 10.1371/journal.pgen.1003232 PMC3554576

[20] Novo M, Bigey F, Beyne E, Galeote V, Gavory F, et al (2009) Eukaryote-to-eukaryote gene transfer events revealed by the genome sequence of the wine yeast Saccharomyces cerevisiae EC1118. Proc Natl Acad Sci U S A 106: 16333–16338. 19805302 10.1073/pnas.0904673106 PMC2740733

[21] Galeote V, Bigey F, Beyne E, Novo M, Legras JL, et al (2011) Amplification of a Zygosaccharomyces bailii DNA segment in wine yeast genomes by extrachromosomal circular DNA formation. PLoS One 6: e17872. 21423766 10.1371/journal.pone.0017872 PMC3053389

[22] Hall C, Brachat S, Dietrich FS (2005) Contribution of Horizontal Gene Transfer to the Evolution of Saccharomyces cerevisiae. Eukaryot Cell 4: 1102–1115. 15947202 10.1128/EC.4.6.1102-1115.2005 PMC1151995

[23] Galeote V, Novo M, Salema-Oom M, Brion C, Valerio E, et al (2010) FSY1, a horizontally transferred gene in the Saccharomyces cerevisiae EC1118 wine yeast strain, encodes a high-affinity fructose/H+ symporter. Microbiology 156: 3754–3761. 20705659 10.1099/mic.0.041673-0

[24] Ness F, Aigle M (1995) RTM1: a member of a new family of telomeric repeated genes in yeast. Genetics 140: 945–956. 7672593 10.1093/genetics/140.3.945 PMC1206678

[25] Chen G, Bradford WD, Seidel CW, Li R (2012) Hsp90 stress potentiates rapid cellular adaptation through induction of aneuploidy. Nature 482: 246–250. 22286062 10.1038/nature10795 PMC3276732

[26] Infante JJ, Dombek KM, Rebordinos L, Cantoral JM, Young ET (2003) Genome-Wide Amplifications Caused by Chromosomal Rearrangements Play a Major Role in the Adaptive Evolution of Natural Yeast. Genetics 165: 1745–1759. 14704163 10.1093/genetics/165.4.1745 PMC1462916

[27] Yu X, Gabriel A (2004) Reciprocal Translocations in Saccharomyces cerevisiae Formed by Nonhomologous End Joining. Genetics 166: 741–751. 15020464 10.1093/genetics/166.2.741 PMC1470746

[28] Pennaneach V, Kolodner RD (2009) Stabilization of Dicentric Translocations through Secondary Rearrangements Mediated by Multiple Mechanisms in *S. cerevisiae* . PLoS ONE 4: e6389. 19636429 10.1371/journal.pone.0006389 PMC2712687

[29] Jackson SP (2002) Sensing and repairing DNA double-strand breaks. Carcinogenesis 23: 687–696. 12016139 10.1093/carcin/23.5.687

[30] Agarwal S, Tafel AA, Kanaar R (2006) DNA double-strand break repair and chromosome translocations. DNA Repair (Amst) 5: 1075–1081. 16798112 10.1016/j.dnarep.2006.05.029

[31] Fischer G, James SA, Roberts IN, Oliver SG, Louis EJ (2000) Chromosomal evolution in *Saccharomyces* . Nature 405: 451–454. 10839539 10.1038/35013058

[32] Greig D (2008) Reproductive isolation in Saccharomyces. Heredity 102: 39–44. 18648383 10.1038/hdy.2008.73

[33] Rachidi N, Barre P, Blondin B (1999) Multiple Ty-mediated chromosomal translocations lead to karyotype changes in a wine strain of Saccharomyces cerevisiae. Mol Gen Genet 261: 841–850. 10394922 10.1007/s004380050028

[34] Umezu K, Hiraoka M, Mori M, Maki H (2002) Structural Analysis of Aberrant Chromosomes That Occur Spontaneously in Diploid Saccharomyces cerevisiae: Retrotransposon Ty1 Plays a Crucial Role in Chromosomal Rearrangements. Genetics 160: 97–110. 11805048 10.1093/genetics/160.1.97 PMC1461932

[35] Bidenne C, Blondin B, Dequin S, Vezinhet F (1992) Analysis of the chromosomal DNA polymorphism of wine strains of *Saccharomyces cerevisiae* . Curr Genet 22: 1–7. 1611665 10.1007/BF00351734

[36] Liti G, Peruffo A, James SA, Roberts IN, Louis EJ (2005) Inferences of evolutionary relationships from a population survey of LTR-retrotransposons and telomeric-associated sequences in the Saccharomyces sensu stricto complex. Yeast 22: 177–192. 15704235 10.1002/yea.1200

[37] Nardi T, Corich V, Giacomini A, Blondin B (2010) A sulphite-inducible form of the sulphite efflux gene SSU1 in a Saccharomyces cerevisiae wine yeast. Microbiology 156: 1686–1696. 20203053 10.1099/mic.0.036723-0

[38] Tosato V, Nicolini C, Bruschi CV (2009) DNA bridging of yeast chromosomes VIII leads to near-reciprocal translocation and loss of heterozygosity with minor cellular defects. Chromosoma 118: 179–191. 19015868 10.1007/s00412-008-0187-z

[39] Yuasa N, Nakagawa Y, Hayakawa M, Iimura Y (2005) Two alleles of the sulfite resistance genes are differentially regulated in Saccharomyces cerevisiae. Biosci Biotechnol Biochem 69: 1584–1588. 16116289 10.1271/bbb.69.1584

[40] Rossi B, Noel P, Bruschi CV (2010) Different aneuploidies arise from the same bridge-induced chromosomal translocation event in Saccharomyces cerevisiae. Genetics 186: 775–790. 20805555 10.1534/genetics.110.120683 PMC2975295

[41] Perez-Ortin JE, Querol A, Puig S, Barrio E (2002) Molecular characterization of a chromosomal rearrangement involved in the adaptive evolution of yeast strains. Genome Res 12: 1533–1539. 12368245 10.1101/gr.436602 PMC187534

[42] Park H, Bakalinsky AT (2000) SSU1 mediates sulphite efflux in Saccharomyces cerevisiae. Yeast 16: 881–888. 10870099 10.1002/1097-0061(200007)16:10<881::AID-YEA576>3.0.CO;2-3

[43] Shibata Y, Malhotra A, Bekiranov S, Dutta A (2009) Yeast genome analysis identifies chromosomal translocation, gene conversion events and several sites of Ty element insertion. Nucleic Acids Research 37: 6454–6465. 19710036 10.1093/nar/gkp650 PMC2770650

[44] Marullo P, Yvert G, Bely M, Aigle M, Dubourdieu D (2007) Efficient use of DNA molecular markers to construct industrial yeast strains. FEMS Yeast Res Vol 7: 1295. 10.1111/j.1567-1364.2007.00281.x 17888000

[45] Li H, Handsaker B, Wysoker A, Fennell T, Ruan J, et al (2009) The Sequence Alignment/Map format and SAMtools. Bioinformatics 25: 2078–2079. 19505943 10.1093/bioinformatics/btp352 PMC2723002

[46] Danecek P, Auton A, Abecasis G, Albers CA, Banks E, et al (2011) The variant call format and VCFtools. Bioinformatics 27: 2156–2158. 21653522 10.1093/bioinformatics/btr330 PMC3137218

[47] Chevreux B, Pfisterer T, Drescher B, Driesel AJ, Müller WE, et al (2004) Using the miraEST assembler for reliable and automated mRNA transcript assembly and SNP detection in sequenced ESTs. Genome research 14: 1147–1159. 15140833 10.1101/gr.1917404 PMC419793

[48] Albertin W, Da Silva T, Rigoulet M, Salins B, Masneuf-Pomarède I, et al (in press) The mitochondrial DNA impacts respiration but not fermentation in inter specific Saccharomyces hybrids. Plos one 10.1371/journal.pone.0075121 PMC3781082 24086452

[49] Gietz RD, Schiestl RH (1991) Applications of high efficiency lithium acetate transformation of intact yeast cells using single-stranded nucleic acids as carrier. Yeast 7: 253–263. 1882550 10.1002/yea.320070307

[50] Teste MA, Duquenne M, Francois JM, Parrou JL (2009) Validation of reference genes for quantitative expression analysis by real-time RT-PCR in *Saccharomyces cerevisiae* . BMC Mol Biol 10: 99. 19874630 10.1186/1471-2199-10-99 PMC2776018

[51] Marullo P, Bely M, Masneuf-Pomarede I, Pons M, Aigle M, et al (2006) Breeding strategies for combining fermentative qualities and reducing off-flavor production in a wine yeast model. FEMS Yeast Res 6: 268–279. 16487348 10.1111/j.1567-1364.2006.00034.x

[52] Pate JB, Lodge JP, Wartburg AF (1962) Effect of Pararosaniline in the Trace Determination of Sulfur Dioxide. Analytical Chemistry 34: 1660–1662.

[53] R Development Core Team (2010) R: A language and environment for statistical computing. Vienna, Austria: R Foundation for Statistical Computing.

[54] Engle EK, Fay JC (2012) Divergence of the yeast transcription factor FZF1 affects sulfite resistance. PLoS Genet 8: e1002763. 22719269 10.1371/journal.pgen.1002763 PMC3375221

[55] Tachibana C, Yoo JY, Tagne JB, Kacherovsky N, Lee TI, et al (2005) Combined global localization analysis and transcriptome data identify genes that are directly coregulated by Adr1 and Cat8. Mol Cell Biol 25: 2138–2146. 15743812 10.1128/MCB.25.6.2138-2146.2005 PMC1061606

[56] Ng PC, Henikoff S (2001) Predicting deleterious amino acid substitutions. Genome Res 11: 863–874. 11337480 10.1101/gr.176601 PMC311071

[57] Goto-Yamamoto N, Kitano K, Shiki K, Yoshida Y, Suzuki T, et al (1998) SSU1-R, a sulfite resistance gene of wine yeast, is an allele of SSU1 with a different upstream sequence. Journal of Fermentation and Bioengineering 86: 427–433.

[58] Yuasa N, Nakagawa Y, Hayakawa M, Iimura Y (2004) Distribution of the sulfite resistance gene SSU1-R and the variation in its promoter region in wine yeasts. J Biosci Bioeng 98: 394–397. 16233727 10.1016/S1389-1723(04)00303-2

[59] Ribéreau-Gayon P, Dubourdieu D, Donèche B, Lonvaud A (2000) Alcoholic fermentation and metabolic pathways. Handbook of Enology Vol1. London: John Wiley & Sons.

[60] Henick K, Edinger, Daniel, Monk (1998) Selective effects of sulfur dioxide and yeast starter culture addition on indigenous yeast populations and sensory characteristics of wine. Journal Of Applied Microbiology 84: 865–876.

[61] Egli CM, Edinger WD, Mitrakul CM, Henick-Kling T (1998) Dynamics of indigenous and inoculated yeast populations and their effect on the sensory character of Riesling and Chardonnay wines. Journal Of Applied Microbiology 85: 779–789. 9830113 10.1046/j.1365-2672.1998.00521.x

[62] Winzeler EA, Richards DR, Conway AR, Goldstein AL, Kalman S, et al (1998) Direct allelic variation scanning of the yeast genome. Science 281: 1194–1197. 9712584 10.1126/science.281.5380.1194

[63] Gresham D, Ruderfer DM, Pratt SC, Schacherer J, Dunham MJ, et al (2006) Genome-wide detection of polymorphisms at nucleotide resolution with a single DNA microarray. Science 311: 1932–1936. 16527929 10.1126/science.1123726

[64] Deutschbauer AM, Davis RW (2005) Quantitative trait loci mapped to single-nucleotide resolution in yeast. Nat Genet 37: 1333–1340. 16273108 10.1038/ng1674

[65] Ambroset C, Petit M, Brion C, Sanchez I, Delobel P, et al (2011) Deciphering the molecular basis of wine yeast fermentation traits using a combined genetic and genomic approach. G3 (Bethesda) 1: 263–281. 22384338 10.1534/g3.111.000422 PMC3276144

[66] Cubillos FA, Billi E, Zorgo E, Parts L, Fargier P, et al (2011) Assessing the complex architecture of polygenic traits in diverged yeast populations. Mol Ecol 20: 1401–1413. 21261765 10.1111/j.1365-294X.2011.05005.x

[67] Bloom JS, Ehrenreich IM, Loo WT, Lite TLV, Kruglyak L (2013) Finding the sources of missing heritability in a yeast cross. Nature 494: 234–237. 23376951 10.1038/nature11867 PMC4001867

[68] Ehrenreich IM, Bloom J, Torabi N, Wang X, Jia Y, et al (2012) Genetic Architecture of Highly Complex Chemical Resistance Traits across Four Yeast Strains. Plos Genetics 8. 10.1371/journal.pgen.1002570 PMC3305394 22438822

[69] Katou T, Namise M, Kitagaki H, Akao T, Shimoi H (2009) QTL mapping of sake brewing characteristics of yeast. J Biosci Bioeng 107: 383–393. 19332297 10.1016/j.jbiosc.2008.12.014

[70] Divol B, du Toit M, Duckitt E (2012) Surviving in the presence of sulphur dioxide: strategies developed by wine yeasts. Appl Microbiol Biotechnol 95: 601–613. 22669635 10.1007/s00253-012-4186-x

[71] Stratford M, Morgan P, Rose A (1987) Sulphur dioxide resistance in Saccharomyces cerevisiae and Saccharomycodes ludwigii. Journal of general microbiology 133: 2173–2179.

[72] Hinze H, Holzer H (1986) Analysis of the energy metabolism after incubation of Saccharomyces cerevisiae with sulfite or nitrite. Archives of microbiology 145: 27–31. 3530169 10.1007/BF00413023

[73] Park H, Hwang YS (2008) Genome-wide transcriptional responses to sulfite in Saccharomyces cerevisiae. J Microbiol 46: 542–548. 18974956 10.1007/s12275-008-0053-y

[74] Thornton RJ (1982) Selective hybridisation of pure culture wine yeasts. European journal of applied microbiology and biotechnology 14: 159–164.

[75] Casalone E, Colella CM, Daly S, Gallori E, Moriani L, et al (1992) Mechanism of resistance to sulphite in Saccharomyces cerevisiae. Curr Genet 22: 435–440. 1473174 10.1007/BF00326407

[76] Thomas D, Surdin-Kerjan Y (1997) Metabolism of sulfur amino acids in Saccharomyces cerevisiae. Microbiol Mol Biol Rev 61: 503–532. 9409150 10.1128/mmbr.61.4.503-532.1997 PMC232622

[77] Xu X, Wightman JD, Geller BL, Avram D, Bakalinsky AT (1994) Isolation and characterization of sulfite mutants of Saccharomyces cerevisiae. Current Genetics 25: 488–496. 8082198 10.1007/BF00351667

[78] Badis G, Chan ET, van Bakel H, Pena-Castillo L, Tillo D, et al (2008) A Library of Yeast Transcription Factor Motifs Reveals a Widespread Function for Rsc3 in Targeting Nucleosome Exclusion at Promoters. Molecular Cell 32: 878–887. 19111667 10.1016/j.molcel.2008.11.020 PMC2743730

[79] Favier M, Bilhère E, Lonvaud-Funel A, Moine V, Lucas PM (2012) Identification of pOENI-1 and Related Plasmids in *Oenococcus oen*i Strains Performing the Malolactic Fermentation in Wine. PLoS ONE 7: e49082. 23139835 10.1371/journal.pone.0049082 PMC3489775

[80] Fraser JA, Huang JC, Pukkila-Worley R, Alspaugh JA, Mitchell TG, et al (2005) Chromosomal translocation and segmental duplication in Cryptococcus neoformans. Eukaryotic cell 4: 401–406. 15701802 10.1128/EC.4.2.401-406.2005 PMC549341

[81] Chuma I, Isobe C, Hotta Y, Ibaragi K, Futamata N, et al (2011) Multiple Translocation of the *AVR-Pita* Effector Gene among Chromosomes of the Rice Blast Fungus *Magnaporthe oryzae* and Related Species. PLoS Pathog 7: e1002147. 21829350 10.1371/journal.ppat.1002147 PMC3145791

[82] Koszul R, Dujon B, Fischer G (2006) Stability of Large Segmental Duplications in the Yeast Genome. Genetics 172: 2211–2222. 16489235 10.1534/genetics.105.048058 PMC1456401

[83] Adams J, Puskas-Rozsa S, Simlar J, Wilke C (1992) Adaptation and major chromosomal changes in populations of Saccharomyces cerevisiae. Current Genetics 22: 13–19. 1611666 10.1007/BF00351736

[84] Dhar R, Sagesser R, Weikert C, Yuan J, Wagner A (2011) Adaptation of Saccharomyces cerevisiae to saline stress through laboratory evolution. J Evol Biol 24: 1135–1153. 21375649 10.1111/j.1420-9101.2011.02249.x

