# Supplementary figures and images for: QTL Dissection of Lag Phase in Wine Fermentation Reveals a New Translocation Responsible for Saccharomyces cerevisiae Adaptation to Sulfite
Source: PLoS One. 2014 Jan 28;9(1):e86298. doi: 10.1371/journal.pone.0086298 (PMC3904918; doi:10.1371/journal.pone.0086298)

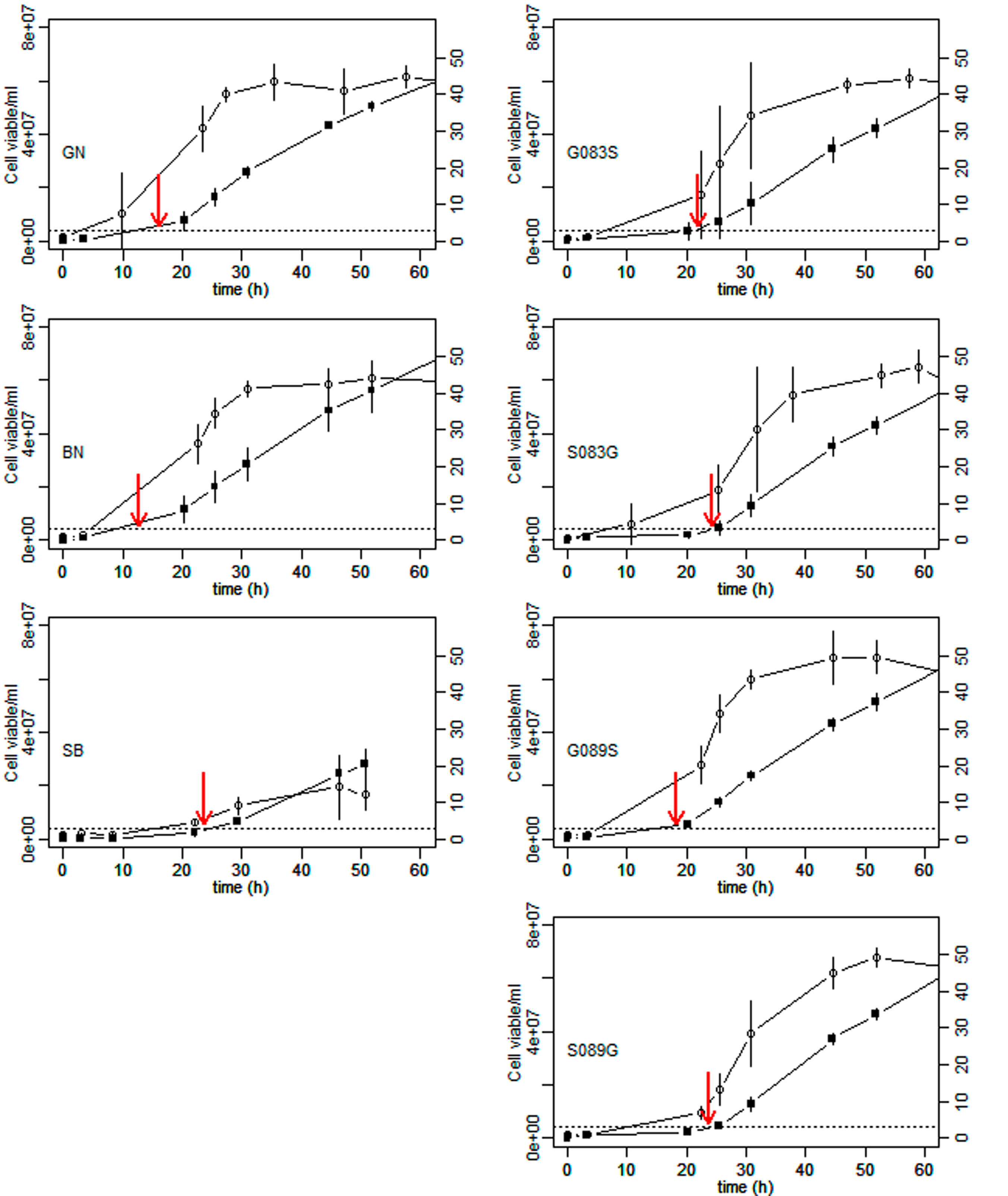

Supplement: Figure S1 — The time course of viable cell concentration (open circle) and the CO2 production (black square) during the 60 first hours after yeast inoculation are shown. Data presented are the mean of three independent repetitions with standard error for parental strains (SB and GN), F1 hybrid (BN) as well as hemizygous hybrids (G083S, S083G, G089S and S089G) for the gene ATG34 (YOL083w) and HAL9 (YOL089c) respectively. The lag phase time computed is indicated by a red arrow. (TIF) [file pone.0086298.s001.tif]

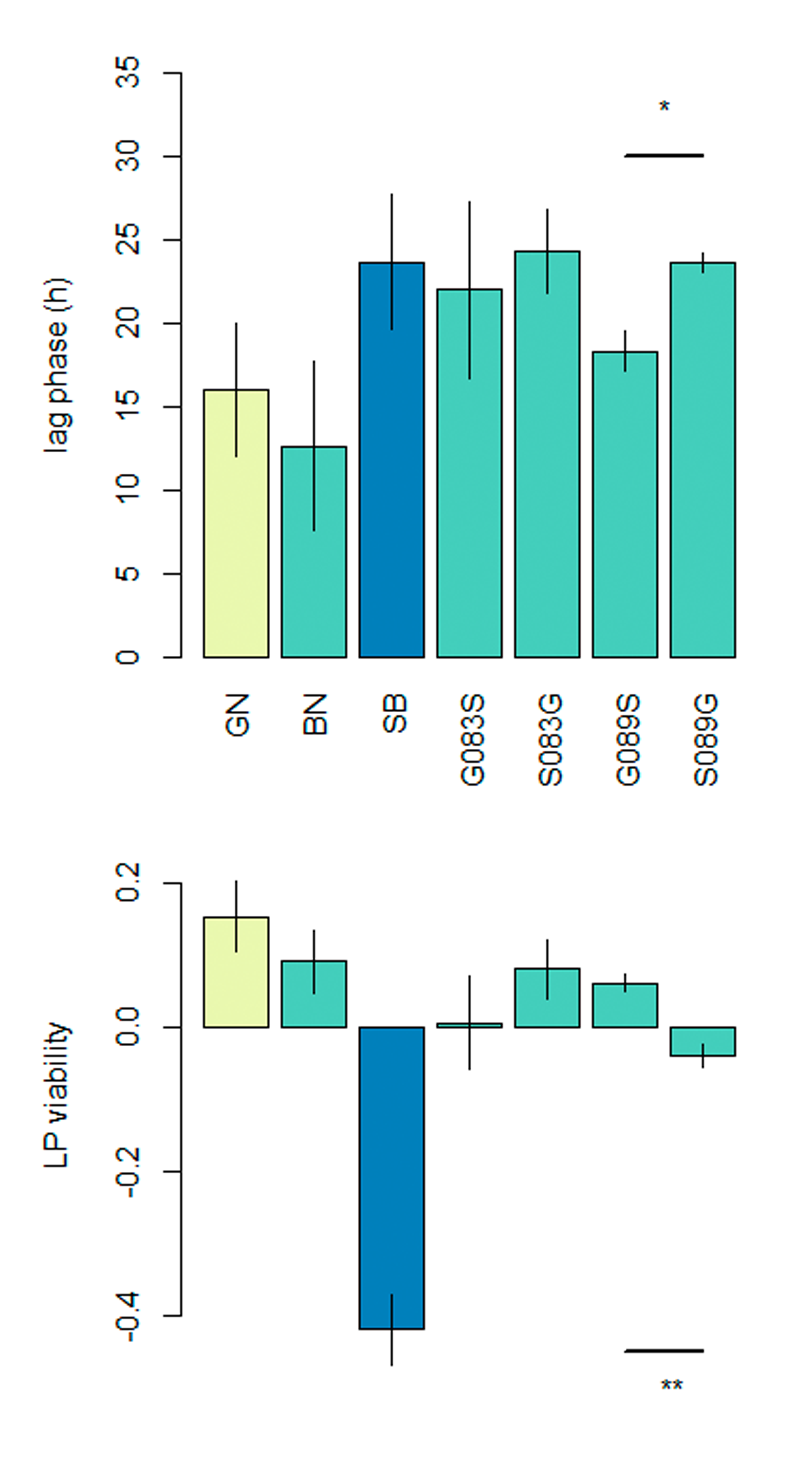

Supplement: Figure S2 — Reciprocal heterozygosity analysis of ATG34 and HAL9 for lag phase. The lag phase (h) as well as the LP viability was measured for parental strains (SB and GN), F1 hybrid (BN) as well as hemizygous hybrids (G083S, S083G, G089S,S089G) for genes ATG34 (YOL083w) and HAL9 (YOL089c). The bar plots represent mean values and standard errors measured for 3 independent repetitions. The statistic difference between isogenic strains was tested using a Wilcoxon test. Isogenic strains are represented with the same color, GN = light green, BN = green, SB = blue. The significant levels was coded *, and **, for p-values<0.05 and <0.01 respectively. (TIF) [file pone.0086298.s002.tif]
