# Supplementary material for: QTL Dissection of Lag Phase in Wine Fermentation Reveals a New Translocation Responsible for Saccharomyces cerevisiae Adaptation to Sulfite
Source: PLoS One. 2014 Jan 28;9(1):e86298. doi: 10.1371/journal.pone.0086298 (PMC3904918; doi:10.1371/journal.pone.0086298)
Supplement: Table S1 — (DOCX) [file pone.0086298.s003.docx]

**Supplementary Table S1 list of industrial strains used for population survey**

| Strain | Origin | Area/Supplier |
| --- | --- | --- |
| Hirondelle | Bakery | Lesaffre |
| Levante | Bakery | Puratos |
| SBA | Bakery | Lesaffre |
| CLIB 650 | Brewery | CLIB |
| Prem. Gold | Brewery | Muntons Etavobra |
| S04 | Brewery | Satale Etavobra |
| US-56 | Brewery | Satale Etavobra |
| NRRL 7327 | Brewery | NRRL |
| 522 Davis | Commercial wine | Laffort |
| BO213 | Commercial wine | Laffort |
| BJL | Commercial wine | Laffort |
| BR115 | Commercial wine | Laffort |
| Exc C1 | Commercial wine | Lamothe Abiet |
| F10 | Commercial wine | Laffort |
| F15 | Commercial wine | Laffort |
| F33 | Commercial wine | Laffort |
| F5 | Commercial wine | Laffort |
| F83 | Commercial wine | Laffort |
| FW | Commercial wine | Lamothe Abiet |
| FX10 | Commercial wine | Laffort |
| RB2 | Commercial wine | Laffort |
| RB4 | Commercial wine | Laffort |
| RMS2 | Commercial wine | Laffort |
| RX60 | Commercial wine | Laffort |
| SP | Commercial wine | Lamothe Abiet |
| SPARK | Commercial wine | Laffort |
| ST | Commercial wine | Laffort |
| VL1 | Commercial wine | Laffort |
| VL2 | Commercial wine | Laffort |
| VL3 | Commercial wine | Laffort |
| X16 | Commercial wine | Laffort |
| XR | Commercial wine | Lamothe Abiet |
| YB-427 | Distillery | NRRL |
| CLIB 294 | Distillery | CLIB |
| A24 | Distillery | Alcotech |
| 12A2 | Grape must/wine | Burgundy |
| 4D2 | Grape must/wine | Burgundy |
| 6A1 | Grape must/wine | Burgundy |
| 8A1 | Grape must/wine | Burgundy |
| F7 2 | Grape must/wine | Sauterne |
| Lg'ld | Grape must/wine | NA |
| LMPR2 | Grape must/wine | Burgundy |
| OLF II | Grape must/wine | Burgundy |
| OLF III | Grape must/wine | Burgundy |
| S328 | Grape must/wine | NA |
| OS104 | Nature | Oak exudate |
| S67 | Grape must/wine | NA |
| XMC30 | Grape must/wine | NA |
