# Supplementary material for: QTL Dissection of Lag Phase in Wine Fermentation Reveals a New Translocation Responsible for Saccharomyces cerevisiae Adaptation to Sulfite
Source: PLoS One. 2014 Jan 28;9(1):e86298. doi: 10.1371/journal.pone.0086298 (PMC3904918; doi:10.1371/journal.pone.0086298)
Supplement: Table S2 — (DOCX) [file pone.0086298.s004.docx]

**Supplementary Table S2 list of primer used**

| Name | Sequence | Description |
| --- | --- | --- |
| p25 | TGGTTTACGAAATGATCCACG | Amplification cassette *HO::KanMx4 or HO::NatMx4* forward |
| p26 | AAATCGAAGACCCATCTGCT | Amplification cassette *HO::KanMx4 or HO::NatMx4*  reverse |
| p732 | TAAGTTCGATCCGTTTGGCGT | insertion test *HO::KanMx4,HO::NatMx4* forward |
| p494 | CATTCGTCTACAATTGAGCC | Amplification cassette *HAL9::KanMx4* forward |
| p495 | CAAAGTTGAGCCTGGTATTTC | Amplification cassette *HAL9::KanMx4* reverse |
| P518 | TTCCTTGAAGTTAACTGTGAA | Insertion test *HAL9::KanMx4* forward |
| p189 | CAAATTGTATTTTAGGAACCG | Deletion test *HAL9* forward |
| p190 | TGATGCCTTGAAACAGCTGTA | Deletion test *HAL9* reverse |
| p500 | AAGAGGCTAGGTCTAATGGCT | Amplification cassette *ATG34::KanMx4* forward |
| p501 | CTTCATTCGTTCTCATTACCC | Amplification cassette *ATG34::KanMx4* reverse |
| p726 | CCTCCGGATGTTTTGGAAACC | Insertion test *ATG34::KanMx4* forward |
| p118 | CGAGTGTTCAGTTACAGGAGG | Deletion test *ATG34* forward |
| p119 | TGCTTGATTCATCAGGGGAA | Deletion test *ATG34* reverse |
| p990 | TCCGCATTTAGACAACACACA | Amplification cassette *SSU1::KanMx4* forward |
| p991 | AGAAGCAAAAGCAGCAAAGC | Amplification cassette *SSU1::KanMx4* reverse |
| p992 | AAGAATAGCCAACCAGCGTGT | Insertion test *SSU1::KanMx4* forward |
| p993 | ATGGCGGCGTTAGTATCGAAT | Insertion test  *SSU1::KanMx4* reverse |
| p988 | ATGGTTGCCAATTGGGTACT | Deletion test *SSU1* forward |
| p989 | AACGCGTAAAATCTAGAGCCG | Deletion test *SSU1* reverse |
| p560 | CGGCGCAGGAACACTG | Insertion test *KanMx4* reverse |
| p733 | ATGCTCATGTAGAGCGCCTG | Insertion test *NatMx4* reverse |
| p605 | CACGGATAGTGGCTTTGGTGAACAATTAC | Q-PCR for *ALG9* gene forward |
| p606 | TATGATTATCTGGCAGCAGGAAAGAACTTGGG | Q-PCR for *ALG9* gene reverse |
| P324 | CACTGGTATTGTTTTGGATACC | Q-PCR for *ACT1* gene reverse |
| p766 | TTTGCGTTTGTTGGTCAATTCTATGCCTTTTA | Q-PCR for *SSU1* gene forward |
| p767 | TCCACGCTTTCAATGCTGTTATACGGAGAA | Q-PCR for *SSU1* gene reverse |
| P323 | TACCGGCCAAATCGATTCTC | Q-PCR for *ACT1* gene forward |
| P904 | TACTGGTATTGTTTTGGATTCC | Q-PCR for *ACT1* gene reverse |
| P758 | AAAGAAGTTGCATGCGCCTA | Translocation XV-t-XVI forward |
| P761 | GAGTTTTTTGCGCCTGCATT | Translocation XVI-t-XV reverse |
| P760 | GCCCCTCCATGTTCTACTATT | Forward Chr XVI avant la cassure de la translocation |
| p761 | AATGCAGGCGCAAAAAACTC | Reverse Chr XV après cassure de la translocation |
| P762 | ACCTATCGAGTCTCCCAC | Forward Chr XVI before break point (Perez-Ortin SSU1MD) |
| P763 | CCATATTTGTGATGATATCG | Reverse Chr VIII after break point (Perez-Ortin EMC34R) |
| P764 | TCGAACATCGAGCATGCA | Forward Chr VIII before break point (Perez-Ortin EMC34D) |
| P765 | GACACCCATGACCATCAC | Reverse Chr XVI after break point (Perez-Ortin SSU1R) |
| P758 | AAAGAAGTTGCATGCGCCTA | Forward Chr XV before break point |
| P765 | GACACCCATGACCATCAC | Reverse Chr XVI after break point (Perez-Ortin SSU1R) |
| P786 | CGCATCCAGTACAAAGAAATG | Forward Chr VIII wt |
| P787 | CTGAGTGATTTGTTTCCCGA | Reverse Chr VIII wt |
| P788 | TCTTTTTGGGCTGGTAGGAT | Forward Chr XVI wt |
| P789 | ATATTTGTAGTGCCTGCACA | Reverse Chr XVI wt |
| p1031 | CATGTTAGGGAGACCAACGA | Forward Chr XV before breakpoint for Δ SSU1 |
| p1032 | GGGGAAAGCTGTAATTTGTGT | Reverse chr XVI after breakpoint for Δ SSU1 |
| P788 | TCTTTTTGGGCTGGTAGGAT | Forward Chr XVI before breakpoint |
| p1032 | GGGGAAAGCTGTAATTTGTGT | Reverse Chr XVI after breakpoint for Δ SSU1 |
