# Supplementary material for: QTL Dissection of Lag Phase in Wine Fermentation Reveals a New Translocation Responsible for Saccharomyces cerevisiae Adaptation to Sulfite
Source: PLoS One. 2014 Jan 28;9(1):e86298. doi: 10.1371/journal.pone.0086298 (PMC3904918; doi:10.1371/journal.pone.0086298)
Supplement: Table S3 — a The SNPs and SAPs presented are given according to the reference genome (S288c strain) http://www.yeastgenome.org. b SIFT analysis was carried out on the web site http://sift.jcvi.org/ using homologous proteins identified by blastp http://blast.ncbi.nlm.nih.gov; the number of proteins aligned varied between 6 to 35 according to the protein tested with a pvalue cut off equal to 0.01. (DOCX) [file pone.0086298.s005.docx]

**Supplementary Table S3 SNP and SAP detected between parental strains in the QTL-XV**

| Strain | SNP ^a^ | Position | Localization | Protein | SAP ^a^ | Amino acid position | SIFT prediction ^b^ |
| --- | --- | --- | --- | --- | --- | --- | --- |
| SB | A -> G | 150503 | *YOL089C* | *HAL9* |  |  |  |
| SB | C -> T | 150605 | *YOL089C* | *HAL9* |  |  |  |
| SB | A -> G | 150635 | *YOL089C* | *HAL9* |  |  |  |
| GN | G -> A | 150771 | *YOL089C* | *HAL9* | S -> L | 907 | Tolerate |
| SB | T -> G | 151024 | *YOL089C* | *HAL9* | I -> L | 823 | Tolerate |
| SB | C -> T | 151025 | *YOL089C* | *HAL9* |  |  |  |
| SB | T -> C | 151073 | *YOL089C* | *HAL9* |  |  |  |
| GN | C -> T | 151326 | *YOL089C* | *HAL9* | G -> D | 722 | Tolerate |
| SB | T->G | 153558 | *YOL089C-YOL088C* |  |  |  |  |
| GN | A -> T | 155115 | *YOL087C* | *DUF1* |  |  |  |
| GN | A -> G | 155438 | *YOL087C* | *DUF1* | L -> P | 1067 | ns |
| GN | G -> A | 155507 | *YOL087C* | *DUF1* | A -> V | 1044 | ns |
| GN | C -> A | 155539 | *YOL087C* | *DUF1* | M -> I | 1033 | ns |
| GN | C -> A | 155997 | *YOL087C* | *DUF1* | L -> V | 881 | ns |
| GN | T -> C | 156018 | *YOL087C* | *DUF1* | E -> K | 874 | ns |
| SB | C -> T | 156099 | *YOL087C* | *DUF1* | G -> S | 847 | ns |
| SB | A -> G | 156784 | *YOL087C* | *DUF1* |  |  |  |
| GN | T -> C | 158329 | *YOL087C* | *DUF1* |  |  |  |
| GN | T->C | 159162 | *YOL087C-YOL086W-A* |  |  |  |  |
| GN | A -> G | 162506 | *YOL084W* | *PHM7* | I --> V | 50 | Tolerate |
| GN | G -> C | 162610 | *YOL084W* | *PHM7* |  |  |  |
| SB | G -> A | 163078 | *YOL084W* | *PHM7* |  |  |  |
| GN | A -> G | 164731 | *YOL084W* | *PHM7* |  |  |  |
| SB | G->C | 165536 | *YOL084W-YOL083W-A* |  |  |  |  |
| SB | T-> A | 166049 | *YOL083W* | *ATG34* |  |  |  |
| GN | G -> C | 166473 | *YOL083W* | *ATG34* | E -> Q | 253 | Tolerate |
| GN | C -> A | 166602 | *YOL083W* | *ATG34* | H -> N | 296 | non Tolerate |
| SB | A -> C | 166822 | *YOL083W* | *ATG34* |  |  |  |
| SB | C -> G | 166851 | *YOL083W* | *ATG34* |  |  |  |
| SB | C -> A | 166852 | *YOL083W* | *ATG34* | C -> A | 379 |  |
| SB | T -> C | 168815 | *YOL082W* | *ATG19* | L -> P | 29 | Tolerate |
| SB | C -> G | 169721 | *YOL082W* | *ATG19* | A -> G | 331 | Tolerate |
| GN | T->C | 170413 | *YOL082W-YOL081* |  |  |  |  |
