# Supplementary material for: QTL Dissection of Lag Phase in Wine Fermentation Reveals a New Translocation Responsible for Saccharomyces cerevisiae Adaptation to Sulfite
Source: PLoS One. 2014 Jan 28;9(1):e86298. doi: 10.1371/journal.pone.0086298 (PMC3904918; doi:10.1371/journal.pone.0086298)
Supplement: Table S4 — Reads were done in pairs. See Materials and Methods for description of the assembly process. Coverage was computed on the basis of the S288c genome size (12071326 nt), obtained from Saccharomyces Genome Database. (DOCX) [file pone.0086298.s006.docx]

**Supplementary Table S4: Overall assembly statistics**

| strain | nb reads | read coverage | nb contigs | contig coverage | assembly size | N50 | N90 |  |
| --- | --- | --- | --- | --- | --- | --- | --- | --- |
| GN | 2039188 | 12.7 | 398 | 0.94 | 11292297 | 50197 | 14308 |  |
| SB | 2790096 | 17.3 | 360 | 0.91 | 11011390 | 67760 | 17641 |  |
